# Skin Barrier Enhancement and Moisturizing Effects of Exosome Extracts Derived from *Pinus densiflora*, *Zanthoxylum piperitum*, and *Lagerstroemia indica* Plants

**DOI:** 10.3390/biology15030249

**Published:** 2026-01-29

**Authors:** Ha-Rim Kim, Seung-Hyeon Lee, Won Been Bae, Min-Ji Shin, Seon-Young Kim, Youn Ok Jung, Mi Hee Park

**Affiliations:** 1 Jeonju AgroBio-Materials Institute, Wonjangdong-gil 111-27, Jeonju 54810, Republic of Korea; poshrim@jami.re.kr (H.-R.K.); sh94@jami.re.kr (S.-H.L.); alswl4101@jami.re.kr (M.-J.S.); seon02@jami.re.kr (S.-Y.K.); 2 Research and Development, Sooy-K Bio Lab, Seongnam 13216, Republic of Korea; rnd10@sooy-k.com

**Keywords:** exosome, skin barrier, moisturizing effect, *Zanthoxylum piperitum*, *Lagerstroemia indica*, *Pinus densiflora*

## Abstract

Exosomes are nano-sized particles with a structure similar to cells, and they are attracting attention as a premium cosmetic raw material because they can be effectively absorbed through skin pores and delivered without decomposing the active ingredients. In this study, the effects of exosome extracts derived from *Zanthoxylum piperitum*, *Lagerstroemia indica*, and *Pinus densiflora* on skin barrier enhancement and moisturizing were evaluated using HaCaT cells. Cell viability was confirmed through MTS assay, and the skin barrier improvement effect was evaluated by analyzing interleukin (IL)-6 expression in an inflammatory response induced by TNFα/IFN-γ. In addition, procollagen, matrix metalloproteinase (MMP)-1, hyaluronic acid, collagenase inhibitory activity, and elastase inhibitory activity were measured to verify the moisturizing effect. The results of the study show that exosome treatment did not affect the viability of HaCaT cells, and the skin barrier improvement effect was confirmed by decreasing IL-6 expression, which increased due to TNF-α/IFN-γ treatment. In addition, after exosome treatment, the expression of procollagen and hyaluronic acid increased, the expression of MMP-1 decreased, and significant improvements in collagenase and elastase inhibitory activities were observed, suggesting a skin moisturizing effect. The results of this study indicate that exosome extracts derived from *Pinus densiflora*, *Zanthoxylum piperitum*, and *Lagerstroemia indica* can contribute to enhancing the skin barrier and moisturizing, providing basic data for the development of exosome-based cosmetic raw materials.

## Simple Summary

This study investigated the effects of exosome extracts derived from *Zanthoxylum piperitum*, *Lagerstroemia indica*, and *Pinus densiflora* on skin barrier improvement and moisturizing using HaCaT cells. Exosome treatment reduced inflammatory IL-6 expression and enhanced procollagen and hyaluronic acid production while inhibiting MMP-1, collagenase, and elastase activities. These findings suggest that plant-derived exosomes can effectively strengthen the skin barrier and improve skin hydration, supporting their potential use in cosmetic development.

## 1. Introduction

The skin, which serves as the body’s largest and most versatile organ, consists of three primary layers, including the epidermis, dermis, and hypodermis. The skin functions as a sophisticated barrier that protects the internal system from the external environment [1,2]. The epidermal barrier functions as a chemical and physical defense, preventing water loss through the skin and maintaining homeostasis in terrestrial environments [3,4]. The outer stratum corneum is composed of anucleated corneocytes embedded in lipid lamellae rich in ceramides, cholesterol, and fatty acids, which together serve as the principal permeability barrier [5,6]. Tight junctions located mainly in the stratum granulosum provide an additional seal against paracellular penetration [7], acting in concert with the lipid layers to maintain moisture and protect against external stressors [8,9,10,11,12]. When these structural elements are compromised, barrier impairment leads to excessive water evaporation and greater susceptibility to microbial or allergenic invasion [13].

Abnormal or excessive immune responses can interfere with normal skin barrier function and contribute to its impairment [14]. An imbalance between Th1- and Th2-driven cytokine responses contributes to chronic cutaneous inflammation. In particular, elevated levels of Th2-associated interleukins, such as IL-4, IL-6, and IL-13, are closely linked to barrier disruption and persistent inflammation observed in disorders of the skin [15,16]. Excessive production of pro-inflammatory cytokines, such as TNF-α and IFN-γ, can hinder keratinocyte maturation, reduce the expression of tight junction components, and disturb normal lipid metabolism [17]. Traditional therapies, including the use of ceramide-rich moisturizers, have shown limited success in repairing the skin barrier, highlighting the need for balanced structural and biochemical regulation to maintain skin homeostasis [18,19].

In recent years, extracellular vesicles (EVs), such as exosomes, have gained attention as innovative bioactive carriers with promising roles in tissue regeneration and dermatological therapy [20]. Exosomes are tiny, membrane-enclosed vesicles, generally 30–150 nm in size, secreted by various cell types. They contain diverse biomolecules, including proteins, lipids, and nucleic acids, that play crucial roles in mediating communication between cells [21,22]. In contrast to artificial nanoparticles, exosomes naturally exhibit high biocompatibility and structural stability, allowing effective delivery of bioactive substances while minimizing immune reactions [23]. Exosomes derived from plants have recently attracted growing interest in cosmetics due to their natural origin and diverse biological activities [24]. Studies have shown that plant-derived exosomes can stimulate collagen production, alleviate oxidative damage, and support skin regeneration [25]. These vesicles present a potential natural substitute for synthetic ingredients in products aimed at anti-aging and strengthening the skin barrier [26]. Although increasing research highlights their therapeutic promise, the precise functions of plant-derived exosomes in preserving or repairing the epidermal barrier remain insufficiently explored.

In the present study, we investigated the protective and restorative effects of exosome extracts isolated from *Pinus densiflora* leaves, *Zanthoxylum piperitum* fruits, and *Lagerstroemia indica* flowers on human keratinocytes. We focused on evaluating their ability to modulate cytokine-induced barrier disruption and to promote the expression of key skin barrier- and moisturization-related biomarkers. Despite growing interest in exosome-based cosmetic materials, only limited information is available regarding the biological activities of plant-derived exosomes, particularly those obtained from *Zanthoxylum piperitum*, *Lagerstroemia indica*, and *Pinus densiflora*. In this work, we comparatively investigated the skin barrier-protective and moisturizing properties of exosomes extracted from these three botanical sources. To our knowledge, this is the first study to systematically evaluate their effects on epidermal inflammation, extracellular matrix regulation, and hydration-related pathways in human keratinocytes. These findings are expected to provide novel insights into the potential application of plant-derived exosomes as innovative cosmetic ingredients.

## 2. Materials and Methods

### 2.1. Isolation and Purification of Exosomes

Crape myrtle (*Lagerstroemia indica*) flowers, Japanese pepper (*Zanthoxylum piperitum*) fruits, and pine (*Pinus densiflora*) needles were each homogenized in triple-distilled water. After mesh filtration, the homogenates were sequentially centrifuged at 4 °C (3500× *g* for 15 min → 15,000× *g* for 30 min → 23,000× *g* for 45 min) to separate the supernatant. The collected supernatant was filtered through a 0.45 µm membrane filter (Hyundai Micro, Seoul, Republic of Korea). The filtrate was then concentrated and purified tenfold using a tangential flow filtration (TFF) system equipped with a 300 kDa membrane (Minimate TFF capsule 300K OMEGA membrane, Pall Corporation, Port Washington, NY, USA). The concentrated solution was further filtered through a 0.2 µm membrane filter (Hyundai Micro, Republic of Korea), and the exosome preparations from each plant source were mixed at a 1:1:1 ratio for subsequent experiments. The prepared exosome samples were stored at −80 °C until use.

### 2.2. Cell Culture

To evaluate the effect of exosome, we used the human keratinocyte HaCaT cells (Cell Line Service GmBH, Eppelheim, Germany). The cells were cultured in Dulbecco’s modified eagle’s medium (DMEM) (Invitrogen, Carlsbad, CA, USA) containing 10% fetal bovine serum (FBS) (Invitrogen, Carlsbad, CA, USA) and penicillin–streptomycin sulfate (100 units/mL and 100 µg/mL) (Invitrogen, Carlsbad, CA, USA) at 37 °C in 5% CO_2_ atmosphere. The cells were maintained at culture densities below 1 × 10^5^ cells/mL.

### 2.3. Cytotoxicity Assay

HaCaT cells in the exponential growth phase were seeded at 1 × 10^5^ cells /well in 96-well culture plates and treated with different doses of exosomes (1 to 1000 µg/mL) for 24 h. Then, the cells were estimated using MTS ([3-(4,5-dimethylthiazol-2-yl)-5-(3-carboxymethoxyphenyl)-2-(4-sulfophenyl)-2H-tetrazolium, inner salt) (Promega, Madison, WI, USA). Ten microliters of MTS solution was added to each well, and the cells were incubated for an additional 4 h. The optical density at 490 nm was measured by a microplate spectrophotometer (Multiskan Go, Thermo Scientific, Waltham, MA, USA). The control cells were considered to be 100% viable. We selected a low experimental dose of exosomes.

### 2.4. Biomarker Analysis Using ELISA

HaCaT cells were seeded into 6-well plates at a density of 5 × 10^5^ cells/mL and cultured. After replacing the fresh medium containing various concentrations of the test samples, the cells were incubated for 24 h. The collected supernatants were then used to evaluate skin-moisturizing efficacy. Inflammatory cytokine IL-6 was quantified using ELISA kits (cat# D6050B; R&D systems, Minneapolis, UK). Biomarkers for moisturizing activity, including type I procollagen (cat# ab210966; Abcam, Cambridge, UK), matrix metalloproteinase (MMP)-1 (cat# ab215083; Abcam, Cambridge, UK), hyaluronan (cat# DHYAL0; R&D systems, Minneapolis, UK), collagenase (cat# E12055; Invitrogen, Waltham, MA, USA), and elastase (cat# E12056; Invitrogen, Waltham, MA, USA) were quantified using ELISA kits.

### 2.5. Western Blot Analysis

Whole-cell lysates of the cultured cells were obtained and separated using sodium dodecyl sulfate–polyacrylamide gel electrophoresis (SDS–PAGE), and Western blot analysis was performed as described previously [27]. We purchased the primary antibodies, including anti-ASAH1 (cat# ab282276), anti-SHMT1 (cat# ab186130), anti-SPTLC1 (cat# ab176706), anti-Involucrin (cat# ab181980), anti-PPAR-α (cat# ab3484), anti-Elastin (cat# ab213720), and anti-HAS-2 (cat# ab140671) from Abcam (Cambridge, UK). Anti-Filaggrin (cat# PA5-116911) was purchased from Thermo Fisher Scientific, Inc. (Waltham, MA, USA), anti-HAS-3 (cat# sc-365322) was purchased from Santa Cruz (Dallas, TX, USA), and anti-p53 (cat# 5254) and anti-COL-1 (cat# 72026) were purchased from Cell Signaling Technology, Inc. (Beverly, MA, USA).

### 2.6. TEM Analysis

The morphological characteristics of the samples were examined using a transmission electron microscope (TEM; H-7600, Hitachi, Japan) operated at an accelerating voltage of 80 kV. For TEM preparation, a 10 µL aliquot of the particle suspension was placed onto a formvar/carbon-coated copper grid (FCF150-CU, 150 mesh) and allowed to adsorb for approximately 1 min. Excess liquid was removed with filter paper, after which the grid was subjected to negative staining with 2% (*w*/*v*) uranyl acetate for 30 s. The stained grids were air-dried completely at room temperature and subsequently imaged at various magnifications to assess particle size and morphology.

### 2.7. NTA Analysis

Particle size distribution and concentration were determined using a nanoparticle tracking analysis system (NTA; ZetaView PMX-120, Particle Metrix, Inning am Ammersee, Germany). Prior to measurement, each sample was diluted with triple-distilled water to obtain an appropriate particle concentration for analysis. The diluted suspension was then introduced into the instrument, and videos were recorded at 11 predefined positions with three consecutive scanning cycles under identical camera settings. The camera sensitivity was set to 85, and the shutter speed was fixed at 100. All recorded videos were processed using the ZetaView software (version 8.05.16 SP7) to calculate the mean particle size, size distribution, and particle concentration.

### 2.8. Lipid Profiling Analysis Using LC-MS/MS

For lipid extraction, 50 µL of PBS and 50 µL of lipid standard solution were added to the pellet sample, followed by tip sonication in an ice bath for 1 min (amplitude 10%, pulse on: 0.5 s, pulse off: 0.5 s). Then, 250 µL of methanol (MeOH) and 1 mL of methyl tert-butyl ether (MTBE) were added, and the mixture was vortexed for 1 h using a rotator. After adding 200 µL of H_2_O, the sample was vortexed again for 10 min on a rotator. Phase separation was achieved by centrifugation at 3000× *g* for 5 min, and the upper layer was transferred to a new Eppendorf tube. To extract residual lipids, 400 µL of MTBE was added to the remaining pellet, vortexed for 10 min, and centrifuged again at 3000× *g* for 5 min. The resulting upper layer was combined with the previously collected supernatant. The pooled extract was completely dried overnight in a SpeedVac and reconstituted in 100 µL of MeOH:CHCl_3_ (9:1, *v*/*v*). An aliquot of 4 µL of each sample was injected into the analytical column of the UPLC system. The samples were separated over 30 min using a linear gradient of solvent B (organic phase) from 1% to 99% at a flow rate of 0.25 mL/min. Each sample was analyzed in triplicate. Lipid identification was performed using LipidSearch version 4.2.21. False-positive peaks were manually filtered from the results to ensure accurate lipid profiling.

### 2.9. Statistical Analysis

The data are expressed as the means ± SDs, and all statistical analyses were performed with Sigmaplot v14.0 software (Systat Software Inc., San Jose, CA, USA). Statistical analysis was applied to identify differences, followed by one-way analysis of variance (ANOVA) and Duncan’s multiple comparison test. A value of *p* < 0.05 was considered significant.

## 3. Results

### 3.1. Characterization of Plant-Derived Exosomes by Transmission Electron Microscopy (TEM) and Nanoparticle Tracking Analysis (NTA)

**Figure 1:**
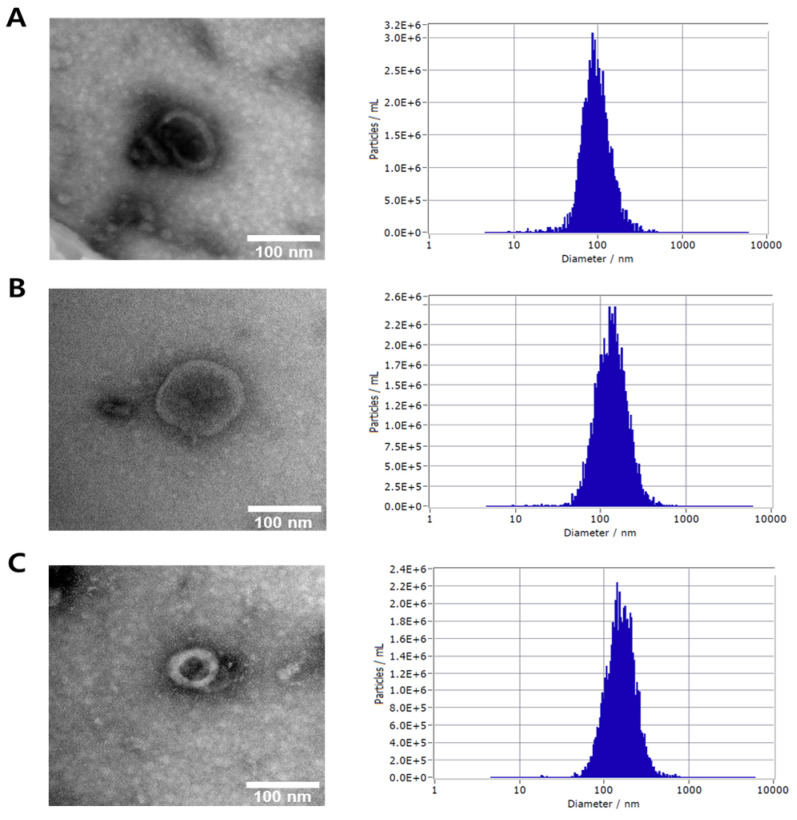
Analysis of exosomes by TEM and NTA. (**A**) TEM (left picture) and NTA (right picture) of exosome from flowers of *Pinus densiflora*. (**B**) TEM (left picture) and NTA (right picture) of exosome from fruits of *Zanthoxylum piperitum*. (**C**) TEM (left picture) and NTA (right picture) of exosome from leaves of *Lagerstroemia indica*.

The morphological and physical characteristics of exosome extracts derived from *Lagerstroemia indica* flowers, *Zanthoxylum piperitum* fruits, and *Pinus densiflora* leaves were analyzed using transmission electron microscopy (TEM) and nanoparticle tracking analysis (NTA). As shown in Figure 1, TEM images revealed that the isolated vesicles exhibited typical cup-shaped or spherical morphologies with intact lipid bilayer membranes, consistent with the known structural features of exosomes. No significant debris or aggregation was observed, indicating that the isolation and purification processes successfully yielded homogeneous vesicle populations. NTA measurements further confirmed the nanoscale size distribution of the exosome preparations. Particle concentration and distribution profiles demonstrated high consistency across all three plant sources, suggesting that the exosomes were stably produced and comparable in their physicochemical properties. These results, together with TEM and NTA analyses, confirm that exosomes derived from *P. densiflora*, *Z. piperitum*, and *L. indica* were successfully isolated and purified, exhibiting uniform morphology and nanoscale size, thereby providing well-characterized vesicles suitable for subsequent biological and functional assays.

### 3.2. Toxicity of Exosome Extracts to Human Cell Lines

**Figure 2:**
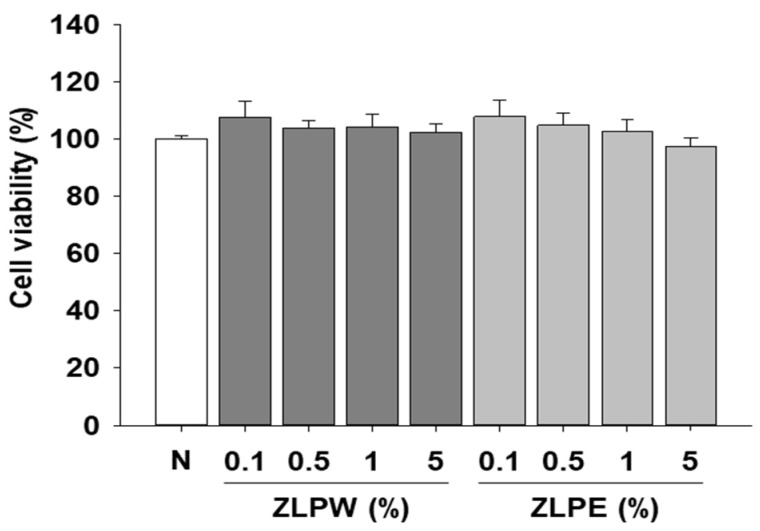
Effects of exosome extracts on cytotoxicity in HaCaT cells. Cells were treated with various doses of exosome extracts for 24 h. Cell viability was assessed using MTS assay to determine the cytotoxicity of exosome extracts (0.1, 0.5, 1, 5%). The results show no significant cytotoxicity at any concentration, confirming safety for use in HaCaT cells. All data are shown as the mean ± SD, with each treatment repeated at least three times.

First, we determined the potential toxicity of exosome to human keratinocyte HaCaT cells by MTS assay. HaCaT keratinocytes were employed in this study because they serve as a widely used epidermal cell model that preserves essential functional traits of primary keratinocytes, such as differentiation potential and sensitivity to external and inflammatory cues. Since the focus of our investigation was on epidermal barrier integrity and keratinocyte-driven inflammatory events, this cell line offered an appropriate experimental system. By contrast, dermal fibroblasts mainly participate in extracellular matrix production and dermal repair processes, and are therefore more relevant to studies centered on dermal remodeling rather than epidermal physiology. Although fibroblast-based experiments could complement our findings, HaCaT cells were the most suitable model for addressing the epidermal aspects examined in this research. The results depicted in Figure 2 clearly show that the exosome extracts did not induce cytotoxicity within the treated concentration range.

### 3.3. Inhibitory Effect of Exosome Extracts on the Level of IL-6 in Human Cell Lines

**Figure 3:**
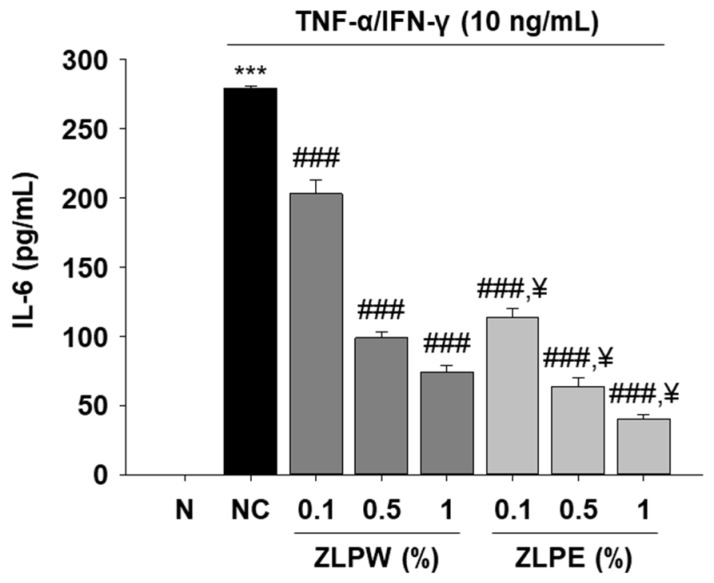
Effects of exosome extracts derived from *Pinus densiflora*, *Zanthoxylum piperitum*, and *Lagerstroemia indica* on skin barrier enhancement in TNF-α/IFN-γ-stimulated HaCaT cells. IL-6 secretion was measured by ELISA after TNF-α/IFN-γ (10 ng/mL) stimulation with or without exosome extract treatment. All data is shown as the mean ± SD, with each treatment repeated at least three times. *** *p* < 0.001 versus N group; ^###^ *p* < 0.001 versus NC group; ^¥^ *p* < 0.001 versus ZLPW group.

To examine the anti-inflammatory effects of plant-derived exosome extracts, IL-6 secretion was evaluated in HaCaT cells following stimulation with TNF-α and IFN-γ (10 ng/mL each). As shown in Figure 3, the cytokine-stimulated group exhibited a marked increase in IL-6 levels compared with the untreated control, confirming successful induction of an inflammatory response. Treatment with exosome extracts significantly reduced IL-6 secretion in a dose-dependent manner, indicating that these vesicles effectively attenuate cytokine-induced inflammation in keratinocytes. Among the tested conditions, exosome-treated groups demonstrated notably greater suppression of IL-6 expression compared to those treated with equivalent concentrations of water extracts (ZLPW group). These findings suggest that exosomes derived from *P. densiflora*, *Z. piperitum*, and *L. indica* exert potent anti-inflammatory activity by modulating the production of pro-inflammatory cytokines, thereby contributing to the maintenance of skin barrier homeostasis under inflammatory stress conditions.

### 3.4. Skin Barrier Enhancement Function of Exosome Extracts in Human Cell Lines

**Figure 4:**
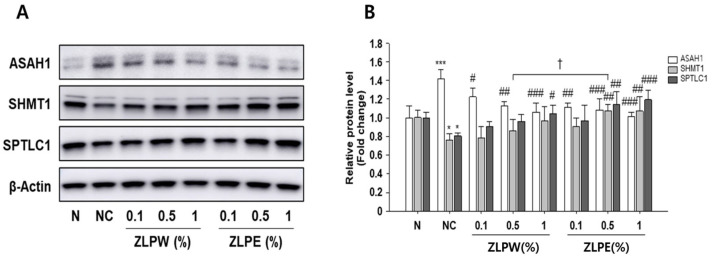
Effects of exosome extracts derived from *Pinus densiflora*, *Zanthoxylum piperitum*, and *Lagerstroemia indica* on skin barrier enhancement in TNF-α/IFN-γ-stimulated HaCaT cells. Protein expression levels (**A**) and relative protein levels (fold change) (**B**) of skin barrier-related markers (ASAH1, SHMT1, and SPTLC1) were analyzed by Western blotting. Equal amounts of total protein were resolved by SDS-PAGE. β-Actin was employed as an internal reference. Western blot analysis was performed three times. All data are shown as the mean ± SD, with each treatment repeated three times. * *p* < 0.05 and *** *p* < 0.001 versus N group; ^#^ *p* < 0.05, ^##^ *p* < 0.01 and ^###^ *p* < 0.001 versus NC group; ^†^ *p* < 0.05 versus ZLPW group.

**Figure 5:**
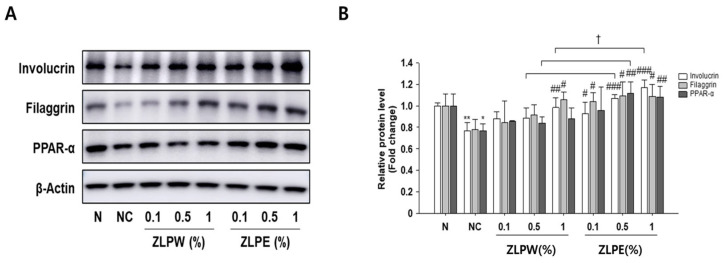
Effects of exosome extracts derived from *Pinus densiflora*, *Zanthoxylum piperitum*, and *Lagerstroemia indica* on skin barrier enhancement in TNF-α/IFN-γ-stimulated HaCaT cells. Protein expression levels (**A**) and relative protein levels (fold change) (**B**) of skin barrier-related markers (involucrin, filaggrin, and PPAR-α) were analyzed by Western blotting. Equal amounts of total protein were resolved by SDS-PAGE. β-Actin was employed as an internal reference. Western blot analysis was performed three times. All data are shown as the mean ± SD, with each treatment repeated three times. * *p* < 0.05 and ** *p* < 0.01 versus N group; ^#^ *p* < 0.05, ^##^ *p* < 0.01 and ^###^ *p* < 0.001 versus NC group; ^†^ *p* < 0.05 versus ZLPW group.

To further clarify the mechanisms underlying the skin barrier-enhancing effects of the plant-derived exosome extracts, the expression of key barrier-associated proteins was analyzed in TNF-α/IFN-γ–stimulated HaCaT cells by Western blotting. As shown in Figure 4 and Figure S1, stimulation with TNF-α/IFN-γ markedly altered the expression of several lipid metabolism and ceramide synthesis-related enzymes, including ASAH1, SHMT1, and SPTLC1, compared with the untreated control. Treatment with the exosome extracts effectively reversed these changes, significantly restoring the expression of SHMT1 and SPTLC1 while reducing ASAH1 levels, which were abnormally elevated under inflammatory conditions. These findings indicate that the exosome extracts help normalize lipid metabolism and ceramide biosynthesis, thereby supporting skin barrier recovery. Additionally, we examined the expression of structural and differentiation-related proteins, including involucrin, filaggrin, and peroxisome proliferator-activated receptor alpha (PPAR-α), which play critical roles in maintaining epidermal integrity (Figure 5 and Figure S1). The results reveal that TNF-α/IFN-γ stimulation substantially decreased the expression of these proteins, whereas treatment with the exosome extracts significantly restored their levels in a dose-dependent manner. Compared to the water extract-treated group (ZLPW), exosome-treated cells showed greater upregulation of these barrier-related proteins.

Collectively, these results demonstrate that exosome extracts derived from *Pinus densiflora*, *Zanthoxylum piperitum*, and *Lagerstroemia indica* promote skin barrier repair by modulating both lipid metabolism-associated enzymes and structural proteins essential for epidermal differentiation and function.

### 3.5. Inhibitory Effect of Exosome Extracts on MMP-1 Secretion and Type I Procollagen Degradation

**Figure 6:**
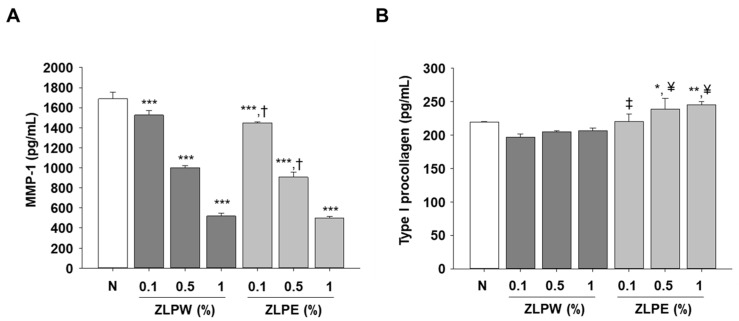
Effects of exosome extracts derived from *Pinus densiflora*, *Zanthoxylum piperitum*, and *Lagerstroemia indica* on moisturizing activity in HaCaT cells. (**A**) MMP-1 secretion was measured by ELISA after treatment with exosome extracts (0.1, 0.5, 1 µg/mL). Exosome treatment significantly reduced MMP-1 levels in a dose-dependent manner, suggesting a protective effect against extracellular matrix degradation. (**B**) Procollagen content was increased with exosome treatment, indicating enhanced collagen synthesis. All data are shown as the mean ± SD, with each treatment repeated at least three times. * *p* < 0.05, ** *p* < 0.01, and *** *p* < 0.001 versus N group; ^†^ *p* < 0.05, ^‡^ *p* < 0.01, and ^¥^ *p* < 0.001 versus ZLPW group.

Type I procollagen is a precursor of type I collagen synthesized in dermal fibroblasts. To investigate whether the plant-derived exosome extracts influenced extracellular matrix maintenance and collagen metabolism, we measured MMP-1 secretion and type I procollagen levels in HaCaT cells after treatment with different concentrations of the exosomes (0.1, 0.5, and 1 µg/mL). As shown in Figure 6A, TNF-α/IFN-γ stimulation markedly increased MMP-1 secretion compared with the untreated control, indicating matrix degradation under inflammatory conditions. Treatment with the exosome extracts significantly reduced MMP-1 levels in a concentration-dependent manner, suggesting that these vesicles exert a protective effect against extracellular matrix breakdown. In contrast, the production of type I procollagen, a key precursor of structural collagen, was substantially enhanced following exosome treatment (Figure 6B). The increase in procollagen levels indicates that the exosomes promoted collagen synthesis and potentially improved dermal matrix remodeling. Furthermore, the exosome-treated groups displayed more pronounced effects than those treated with plant water extracts (ZLPW group), implying superior bioactivity and delivery efficiency of the exosome formulations. These results demonstrate that exosome extracts derived from *P. densiflora*, *Z. piperitum*, and *L. indica* effectively protect skin cells from matrix degradation while stimulating collagen synthesis, contributing to both anti-aging and moisturizing effects.

### 3.6. Effects of Exosome Extracts Derived from Pinus densiflora, Zanthoxylum piperitum, and Lagerstroemia indica on Moisturizing Activity in HaCaT Cells

**Figure 7:**
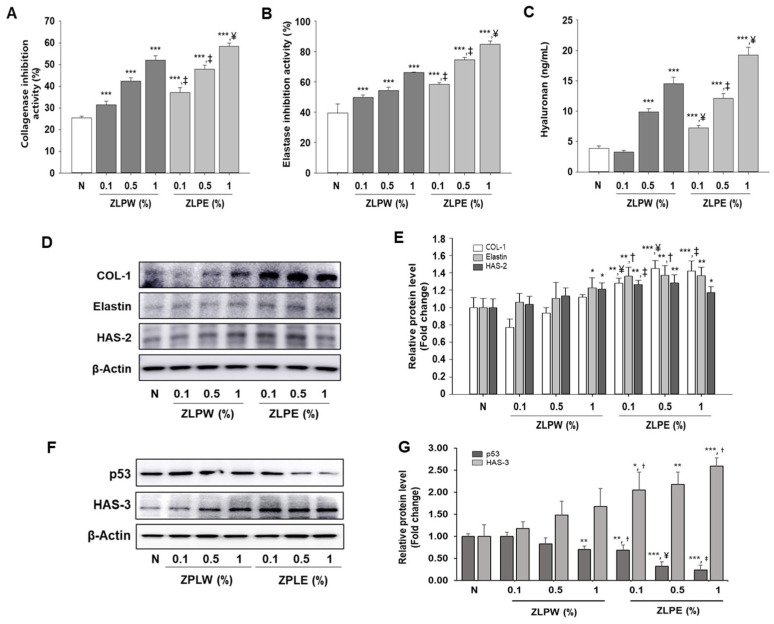
Effects of exosome extracts derived from *Pinus densiflora*, *Zanthoxylum piperitum*, and *Lagerstroemia indica* on moisturizing activity in HaCaT cells. (**A**) Collagenase inhibitory activity and (**B**) elastase inhibitory activity were assessed to evaluate anti-aging effects through the inhibition of skin matrix-degrading enzymes. (**C**) Hyaluronic acid (HA) levels were also elevated, supporting the moisturizing potential of the exosome extracts. Exosome-treated groups showed significantly improved enzyme inhibitory activities. (**D**) Protein expression levels (**E**) and relative protein levels (fold change) of COL-1, elastin, and HAS-2 were analyzed by Western blotting. (**F**) Protein expression levels (**G**) and relative protein levels (fold change) of HAS-3 and p53 were analyzed by Western blotting. Equal amounts of total protein were resolved by SDS-PAGE. β-Actin was employed as an internal reference. Western blot analysis was performed three times. All data are shown as the mean ± SD, with each treatment repeated three times. * *p* < 0.05, ** *p* < 0.01, and *** *p* < 0.001 versus N group; ^†^ *p* < 0.05, ^‡^ *p* < 0.01, and ^¥^ *p* < 0.001 versus ZLPW group.

To investigate the moisturizing and anti-aging effects of exosome extracts derived from *Pinus densiflora*, *Zanthoxylum piperitum*, and *Lagerstroemia indica*, we examined their ability to inhibit extracellular matrix-degrading enzymes and to enhance key skin hydration markers in HaCaT cells. As shown in Figure 7, treatment with the plant-derived exosome extracts significantly suppressed both collagenase (Figure 7A) and elastase (Figure 7B) activities in a concentration-dependent manner compared to the non-treated control group. This inhibitory effect suggests that the exosomes effectively protect the extracellular matrix by preventing collagen and elastin degradation, thereby contributing to skin elasticity maintenance and anti-aging potential. In addition, exosome treatment markedly increased the production of hyaluronic acid (HA) (Figure 7C), a key humectant responsible for maintaining skin hydration [28]. These results indicate that the exosome extracts enhance the skin’s moisture-retaining capacity through both enzymatic inhibition and stimulation of hydrating factors. Furthermore, Western blot analysis confirmed that the expression levels of collagen type I (COL-1), elastin, hyaluronan synthase-2 (HAS-2), and HAS-3 were significantly upregulated in exosome-treated cells compared with the control group (Figure 7D–G and Figure S1). In addition, the expression of p53, a key regulator of apoptosis and cellular senescence, was markedly reduced following exosome treatment (Figure 7F,G and Figure S1). Collectively, these findings indicate that exosome extracts derived from the three plant species exert potent moisturizing and anti-aging effects by enhancing extracellular matrix synthesis and skin hydration while attenuating apoptotic and senescence-associated pathways and suppressing extracellular matrix degradation.

## 4. Discussion

This study revealed that exosome extracts obtained from *Pinus densiflora*, *Zanthoxylum piperitum*, and *Lagerstroemia indica* exhibit multiple positive effects on skin hydration and barrier reinforcement. The combined exosome preparation mitigated TNF-α/IFN-γ-induced inflammation and promoted the expression of key structural proteins and lipid metabolism regulators vital for epidermal balance. Overall, these results suggest that plant-derived exosomes function as natural bio-carriers capable of restoring damaged skin through synergistic anti-inflammatory and regenerative actions.

The decreased IL-6 secretion detected in HaCaT cells implies that the exosome extracts regulate cytokine-dependent inflammatory pathways. Because IL-6 is a major inflammatory mediator known to weaken tight junctions and exacerbate barrier damage [28], its downregulation suggests that these vesicles may contribute to preserving epidermal stability under inflammatory conditions. This immune-regulating effect is consistent with earlier findings showing that plant-derived extracellular vesicles can modulate the MAPK and NF-κB signaling pathways, leading to decreased production of pro-inflammatory cytokines.

Similarly, the increased expression of ASAH1, SHMT1, and SPTLC1 suggests that the exosome extracts contribute to lipid remodeling and ceramide synthesis processes essential for preserving the structural integrity and permeability control of the stratum corneum [29,30]. The restored expression of filaggrin, involucrin, and PPAR-α further indicates improved epidermal differentiation and lipid barrier formation. Together, these findings demonstrate that exosome treatment strengthens barrier architecture, offering sustained support beyond mere suppression of inflammation. Moreover, the exosome extracts enhanced extracellular matrix organization and hydration by promoting the synthesis of type I procollagen and hyaluronic acid, while concurrently inhibiting the activities of MMP-1, collagenase, and elastase. These results indicate that exosomes help prevent extracellular matrix breakdown and enhance dermal elasticity, consistent with their lipid composition enriched in phosphatidylethanolamine (PE), phosphatidylinositol (PI), and ceramide species, as presented in Table 1. The top 30 lipids with the highest average peak areas in the ZPLE samples are summarized in Table 1, and classification of major lipid categories and classes are summarized in Table 2. The relative abundance of each lipid was calculated by dividing the mean MS1 peak area of each lipid by the sum of the mean MS1 peak areas of all detected lipids in each sample and expressing the value as a percentage. In the samples, PE and PI were detected in the highest proportions, in that order. These lipids are recognized for promoting vesicle and cell membrane fusion and regulating signaling pathways associated with skin hydration [31].

The broad spectrum efficacy observed may stem from the distinctive blend of metabolites encapsulated within exosomes derived from the three plant sources. *P. densiflora* is rich in terpenes and polyphenols, *Z. piperitum* contains alkaloids and flavonoids with anti-inflammatory potential, and *L. indica* provides triterpenes and anthocyanins with strong antioxidant properties. The combination of these bioactive compounds likely generate a synergistic effect, enhancing both the stability and functional activity of the exosomes compared to those derived from a single plant source.

Taken together, these results suggest that plant-derived exosomes can serve as eco-friendly, biocompatible alternatives to synthetic nanoparticles for skin health applications. By simultaneously targeting inflammation, lipid metabolism, and extracellular matrix synthesis, they may provide comprehensive protection against environmental stress and aging. Nevertheless, further studies are required to elucidate the precise molecular pathways involved, confirm vesicle uptake mechanisms in vivo, and evaluate formulation stability in cosmetic applications.

## 5. Conclusions

Exosome extracts obtained from *Zanthoxylum piperitum*, *Lagerstroemia indica*, and *Pinus densiflora* were found to strengthen skin barrier function by attenuating inflammation and restoring the expression of key barrier-associated proteins in TNF-α/IFN-γ–stimulated HaCaT cells. Notably, the levels of essential structural and regulatory proteins, including involucrin, filaggrin, PPAR-α, SHMT1, and SPTLC1, were recovered, leading to enhanced epidermal differentiation, lipid metabolism, and ceramide production. In addition, the extracts demonstrated significant moisturizing effects by stimulating the synthesis of procollagen and hyaluronic acid, suppressing MMP-1 expression, and promoting collagenase and elastase inhibition. Collectively, these findings indicate that plant-derived exosomes hold strong potential as cosmetic ingredients for reinforcing skin barrier integrity and maintaining hydration. Their dual functionality in restoring skin architecture and sustaining moisture balance underscores their promise for use in next-generation skincare formulations, warranting further investigation for development as active components in functional cosmetics.

## Figures and Tables

**Table 1:** Lipid profiling in exosome extracts derived from *Pinus densiflora, Zanthoxylum piperitum*, and *Lagerstroemia indica*.

Class	Lipid Molecule	Percentage
PE	PE(20:1_18:2)	10.85%
PI	PI(16:0_18:2)	9.22%
PI	PI(16:0_18:3)	6.17%
PE	PE(16:0_18:2)	5.50%
PE	PE(18:2_18:2)	3.64%
PG	PG(16:0_16:0)	3.24%
PG	PG(16:0_18:1)	2.95%
Hex 1 Cer	Hex 1 Cer(d18:2_16:1)	2.62%
PE	PE(16:0_18:3)	2.43%
TG	TG(16:0_18:2_18:3)	2.27%
PI	PI(16:0_16:0)	1.94%
TG	TG(16:0_18:2_18:2)	1.89%
DG	DG(16:0_18:2)	1.75%
PE	PE(18:3_18:2)	1.45%
TG	TG(18:3_18:2_18:2)	1.44%
PI	PI(16:0_18:1)	1.41%
PE	PE(16:0_18:1)	1.39%
PS	PS(22:0_18:2)	1.37%
PS	PS(20:0_18:2)	1.36%
PE	PE(18:1_18:2)	1.28%
PE	PE(18:1_18:2)	1.27%
DG	DG(18:3_18:3)	1.25%
DG	DG(18:3_18:3)	1.23%
PE	PE(18:0_18:3)	1.21%
TG	TG(18:1_18:2_18:2)	1.16%
TG	TG(16:0_18:2_18:3)	1.12%
TG	TG(16:0_18:1_18:2)	1.10%
LPE	LPE(16:0)	1.03%
TG	TG(18:0_18:2_18:3)	0.96%
TG	TG(16:0_18:1_18:1)	0.87%

**Table 2:** Classification of Major Lipid Categories and Classes.

Lipid Categories	Lipid Classes	Lipid Classes
Glycerolipids	Diacylglycerol (DG)	Triacylglycerol (TG)
Glycerophospholipid	Lysophosphatidylcholine (LPC)	Phosphatidylcholine (PC)
	Lysophosphatidyletanolamine (LPE)	Phosphatidylethanolamine (PE)
	Lysophosphatidic acid (LPA)	Phosphatidic acid (PA)
	Lysophosphatidylglycerol (LPG)	Phosphatidylglycerol (PG)
	Lysophosphatidylinositol (LPI)	Phosphatidylinositol (PI)
	Lysophosphatidylserine (LPS)	Phosphatidylserine (PS)
Sphingolipid	Sphingomyelin (SM)	Ceramide (Cer)

## Data Availability

The data presented in this study are available in this article.

## Supplementary Materials

The following supporting information can be downloaded at https://www.mdpi.com/article/10.3390/biology15030249/s1: Figure S1: Original uncropped Western Blot images; (A) Western Blot image for Figure 4A. (B) Western Blot image for Figure 4A. (C) Western Blot image for Figure 4A. (D) Western Blot image for Figure 4A, Figure 5A and Figure 7D. (E) Western Blot image for Figure 5A. (F) Western Blot image for Figure 5A. (G) Western Blot image for Figure 5A. (H) Western Blot image for Figure 7D. (I) Western Blot image for Figure 7D. (J) Western Blot image for Figure 7D. (K) Western Blot image for Figure 7F. (L) Western Blot image for Figure 7F. (M) Western Blot image for Figure 7F.
