# Supplementary figures and images for: Skin Barrier Enhancement and Moisturizing Effects of Exosome Extracts Derived from Pinus densiflora, Zanthoxylum piperitum, and Lagerstroemia indica Plants
Source: Biology (Basel). 2026 Jan 29;15(3):249. doi: 10.3390/biology15030249 (PMC12896924; doi:10.3390/biology15030249)

A

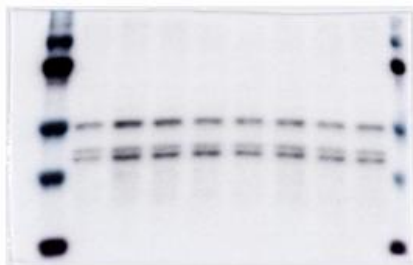

B

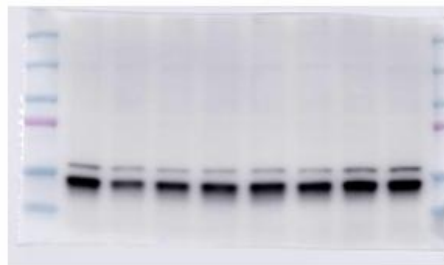

C

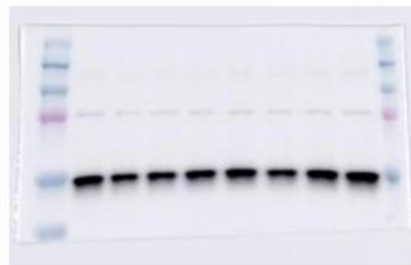

D

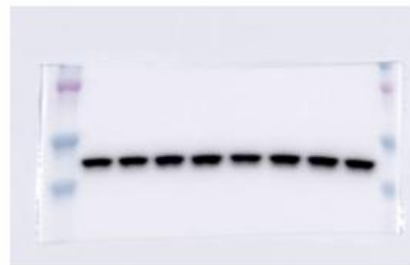

E

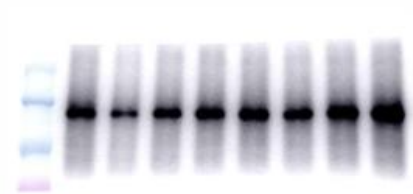

F

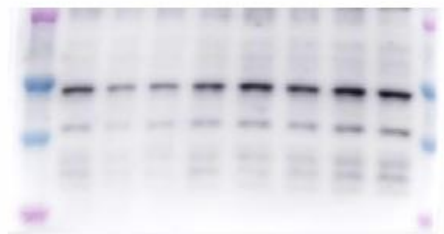

G

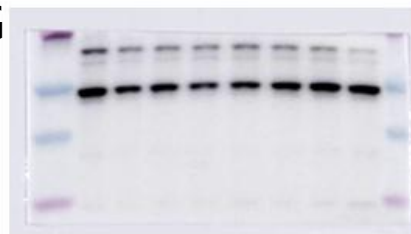

H

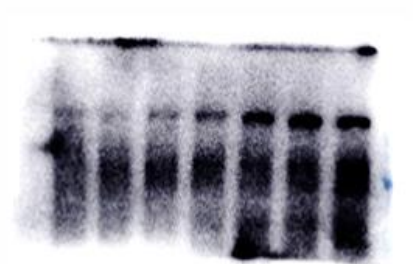

I

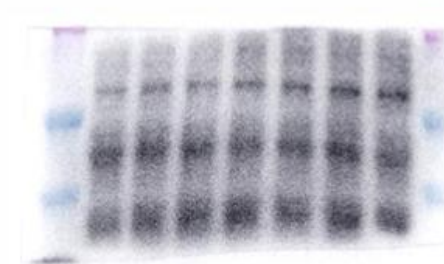

J

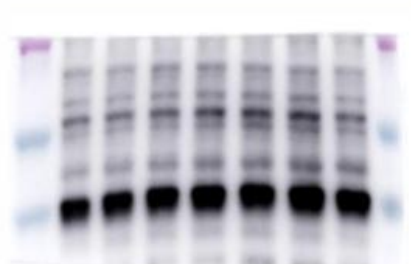

K

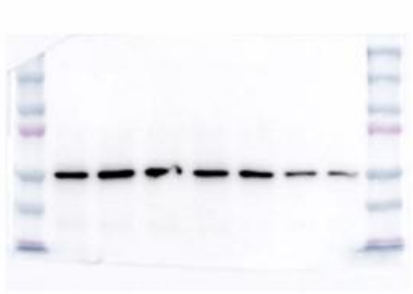

L

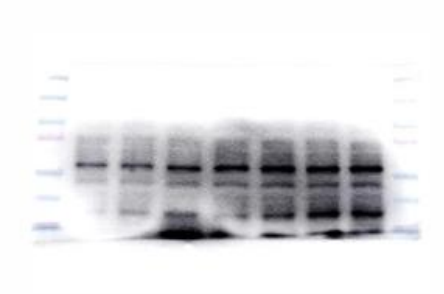

M

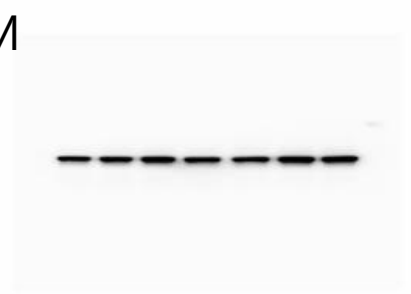

Supplement: Supplementary file 1 [file biology-15-00249-s001.zip › biology-4007514-supplementary.pdf]
